# Deficient methylation and formylation of mt-tRNA^Met^ wobble cytosine in a patient carrying mutations in NSUN3

**DOI:** 10.1038/ncomms12039

**Published:** 2016-06-30

**Authors:** Lindsey Van Haute, Sabine Dietmann, Laura Kremer, Shobbir Hussain, Sarah F. Pearce, Christopher A. Powell, Joanna Rorbach, Rebecca Lantaff, Sandra Blanco, Sascha Sauer, Urania Kotzaeridou, Georg F. Hoffmann, Yasin Memari, Anja Kolb-Kokocinski, Richard Durbin, Johannes A. Mayr, Michaela Frye, Holger Prokisch, Michal Minczuk

**Affiliations:** 1 MRC Mitochondrial Biology Unit, Hills Road, Cambridge CB2 0XY, UK; 2 Wellcome Trust—Medical Research Council Cambridge Stem Cell Institute, Genetics, University of Cambridge, Tennis Court Road, Cambridge CB2 1QR, UK; 3 Helmholtz Zentrum München, Deutsches Forschungszentrum für Gesundheit und Umwelt (GmbH), Institute of Human Genetics, Ingolstädter Landstr. 1, 85764 Neuherberg, Germany; 4 Technical University Munich, Institute of Human Genetics, Trogerstrasse 32, 81675 München, Germany; 5 Department of Biology & Biochemistry, University of Bath, Claverton Down, Bath, BA2 7AY, UK; 6 Department of Physiology Development and Neuroscience, University of Cambridge, Downing Street, Cambridge CB2 3DY, UK; 7 Max-Planck-Institute for Molecular Genetics, Otto-Warburg Laboratory, 14195 Berlin, Germany; 8 University of Würzburg, CU Systems Medicine, 97080 Würzburg, Germany; 9 Max-Delbrück-Center for Molecular Medicine, Berlin Institute for Medical Systems Biology/Berlin Institute of Health, 13125 Berlin, Germany; 10 Department of General Pediatrics, Division of Inherited Metabolic Diseases, University Children's Hospital, 69120 Heidelberg, Germany; 11 Wellcome Trust Sanger Institute, Wellcome Trust Genome Campus, Hinxton CB10 1SA, UK; 12 Department of Paediatrics, Paracelsus Medical University, SALK Salzburg, Salzburg 5020, Austria

## Abstract

Epitranscriptome modifications are required for structure and function of RNA and defects in these pathways have been associated with human disease. Here we identify the RNA target for the previously uncharacterized 5-methylcytosine (m^5^C) methyltransferase NSun3 and link m^5^C RNA modifications with energy metabolism. Using whole-exome sequencing, we identified loss-of-function mutations in *NSUN3* in a patient presenting with combined mitochondrial respiratory chain complex deficiency. Patient-derived fibroblasts exhibit severe defects in mitochondrial translation that can be rescued by exogenous expression of NSun3. We show that NSun3 is required for deposition of m^5^C at the anticodon loop in the mitochondrially encoded transfer RNA methionine (mt-tRNA^Met^). Further, we demonstrate that m^5^C deficiency in mt-tRNA^Met^ results in the lack of 5-formylcytosine (f^5^C) at the same tRNA position. Our findings demonstrate that *NSUN3* is necessary for efficient mitochondrial translation and reveal that f^5^C in human mitochondrial RNA is generated by oxidative processing of m^5^C.

## Abstract (web summary)

The post-transcriptional 5-methylcytosine (m^5^C) modification occurs in a wide range of nuclear-encoded RNAs. Here the authors identify the mitochondrial tRNA-Met as a target for the m^5^C methyltransferase NSun3—found mutated in a mitochondrial disease patient—and link mitochondrial tRNA modifications with energy metabolism.

## Graphical abstract

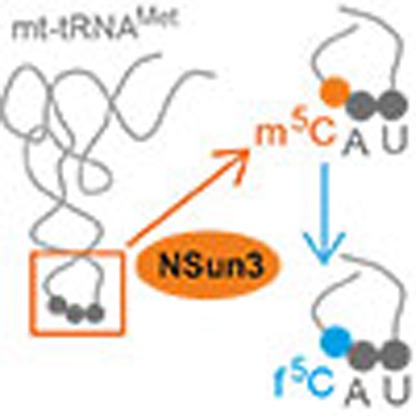

The mitochondrial metabolic pathway of oxidative phosphorylation (OXPHOS) is composed of ∼90 protein components, with 13 of them encoded by the mitochondrial DNA (mtDNA) and translated within the organelle. Defects in mitochondrial function caused by impaired mitochondrial gene expression lead to a wide range of human metabolic diseases. Impaired mitochondrial translation can stem from mtDNA mutations in mitochondrial (mt-) tRNAs or, less frequently, in mt-rRNA (ribosomal RNA). Mutations in nuclear genes coding for enzymes responsible for mtRNA nucleotide modifications, nucleolytic mtRNA processing, proteins involved in mitochondrial ribosome biogenesis and mitochondrial translation factors are also associated with a very rapidly expanding group of human disorders^1^,^2^,^3^.

Of the over 100 known enzyme-catalysed modifications in RNA, methylation is one of the most common^4^. The majority of the methyl-based modifications are conserved from bacteria to mammals and their functions include structural and metabolic stabilization as well as functional roles in regulating protein translation^5^,^6^. The existence of methylated nucleosides in RNA such as 5-methylcytidine (m^5^C) has been described decades ago, but the definition of their precise location, abundance and cellular functions has commenced only recently^7^,^8^,^9^. Mammalian genomes encode at least seven highly conserved RNA:m^5^C methyltransferases of the NOL1/NOP2/Sun (NSun) domain-containing family (NSun 1-7). Human NSun2 and NSun6 both modify cytoplasmic tRNAs. NSun6 methylates position C72 of tRNA^Cys^ and tRNA^Thr^ (ref. 10), while NSun2 has a broader spectrum, methylating various nuclear-encoded tRNAs at positions C34, 48, 49 and 50 (ref. 11, 12) as well as other non-coding RNA species^13^,^14^. Yeast homologue of NSun5 methylates C2278 within a conserved region of the cytosolic large (25S) rRNA and NSun5 orthologues in yeast, worms and flies have been implicated in regulation of lifespan and stress resistance^15^. Mouse NSun4 is localized in mitochondria and methylates small (12S) mitochondrial rRNA, with a role in coordinating mitoribosome assembly^16^.

Here we report mutations in a previously uncharacterized gene, *NSUN3*. We confirm that in humans the protein is localized in mitochondria^17^ and show that it is required for methylation of mt-tRNA^Met^ at the wobble base C34. Furthermore, we demonstrate that NSun3-dependent modification of mt-tRNA^Met^ is a necessary intermediate step towards the formation of 5-formylcytosine (f^5^C) at this position. This study demonstrates that methylation of mt-tRNA^Met^ by NSun3 is an important epitranscriptome modification, necessary for efficient mitochondrial protein synthesis and respiratory function.

## Results

### Loss of NSUN3 in a patient with mitochondrial disease

**Figure 1:**
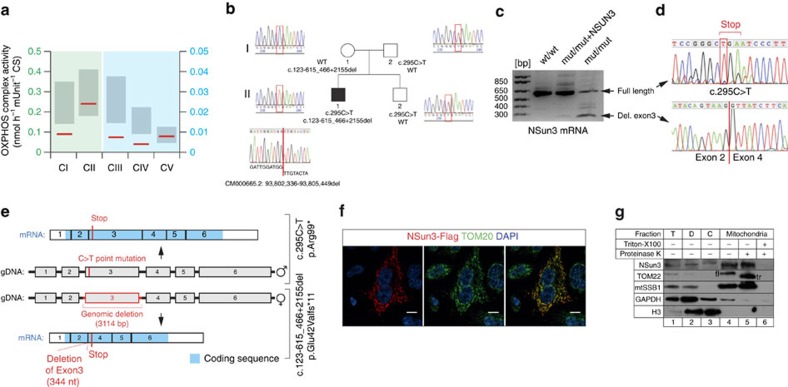
Identification of pathogenic compound heterozygous mutations in *NSun3*. (**a**) OXPHOS complex activity in unfrozen patient muscle homogenate (red line). Grey bars represent the normal range. Note the different scale for complex I–II as compared with complex III–V. (**b**) Segregation analysis and electropherogram corresponding to the genomic DNA mutations identified in the family of a patient carrying NSun3 mutations. The patient's parental allele carries a c.295C>T transition in exon 3, while the maternal allele has a 3,314 nt deletion spanning exon 3 (c.123-615_466+2155del). (**c**) Mutation analysis of NSun3 mRNA level in human dermal fibroblasts of wild-type (wt/wt), patient (mut/mut) and patient cells rescued with a NSUN3 construct (mut/mut+NSUN3). Gel electrophoresis of DNA fragments obtained in a reverse transcriptase–PCR using total RNA from the indicated samples. (**d**) Sanger sequencing of the bands from the mut/mut sample excised from the gel presented in **c** showing a stop mutation in the full-length *NSUN3* mRNA and the lack of exon 3 on the shorter band. (**e**) Schematic overview of the *NSUN3* gene, summarizing the mutations in the patient cells (red) on both genomic DNA (gDNA) and mRNA level. (**f**) Immunofluorescence labelling of a Flag-tagged NSun3 construct (red) in HeLa cells. Cells were counterstained for the mitochondrial import receptor subunit TOM20 (green) and DAPI (blue). Scale bar, 10 μm. (**g**) Sub-cellular localization of NSun3 analysed by western blotting with antibodies against NSun3, TOM22 (mitochondrial outer membrane), mtSSB1 (mitochondrial matrix), GAPDH (cytosol), Histone H3 (nucleus). HEK293T cells were fractionated into debris (‘D', lane 2), cytosol (‘C', lane 3) and mitochondria (‘M', lanes 4–6) ‘T' indicates the total cell lysate. ‘fl' indicates full-length TOM22, ‘tr' stands for truncated TOM22.

We studied a patient recruited in the German network for mitochondrial disorders (mitoNET) who developed mitochondrial disease symptoms at the age of three months (combined developmental disability, microcephaly, failure to thrive, recurrent increased lactate levels in plasma, muscular weakness, proximal accentuated, external ophthalmoplegia and convergence nystagmus) and presented with combined OXPHOS deficiency in skeletal muscle (Fig. 1a, Supplementary Note 1). Using whole-exome sequencing with previously described methodologies and bioinformatic filtering pipelines^18^,^19^, we identified compound heterozygous predicted loss-of-function variants in *NSUN3* (Fig. 1b): (i) a 3,114 bp deletion (c.123-615_466+2155del) removing the entire exon 3 and fragments of the flanking introns, generating aberrant splicing between exons 2 and 4 and a frameshift mutation (p.Glu42Valfs*11; Fig. 1c–e) and (ii) a point mutation (c.295C>T) resulting in a premature stop codon (p.Arg99*) (Fig. 1c–e).

### Mitochondrial localization of NSun3

Since a large-scale proteomic approach has suggested that NSun3 localizes to the mitochondrial matrix^17^ and we detected mitochondrial respiratory chain deficiencies in an individual with potentially pathogenic *NSUN3* variants we set out to confirm NSun3 as a mitochondrial protein. A Flag-tagged version of the NSun3 protein (NSun3-Flag, mRNA GenBank: NM_022072.3), transiently-expressed in HeLa cells, colocalized with the mitochondrial protein TOM20 on immunocytochemistry detection (Fig. 1f). Cellular fractionation experiments of HeLa cells indicated that the endogenous NSun3 is enriched within the mitochondrial fraction, along with a known mitochondrial matrix protein mtSSB1 (Fig. 1g, lane 4). Both NSun3 and mtSSB1 were resistant to proteinase K treatment of the mitochondrial fraction, whereas in similar conditions the outer membrane protein TOM22 was truncated by proteinase K digestion (Fig. 1g, lane 5), confirming that NSun3 is localized within mitochondria in human cells.

### Impaired mitochondrial translation due to NSun3 deficiency

**Figure 2:**
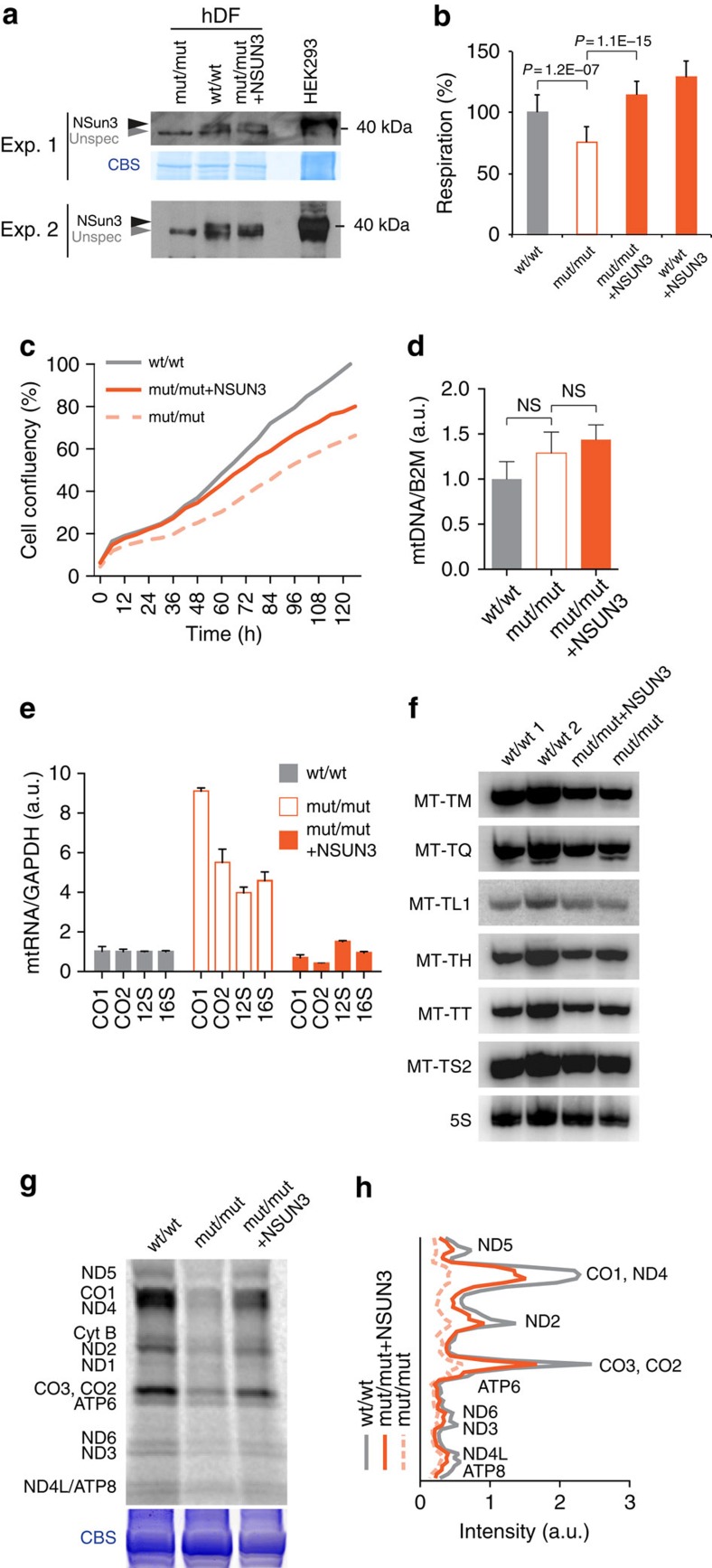
NSun3 is essential for mitochondrial translation. (**a**) Western blot of two different experiments detecting NSun3 protein in mitochondria of human dermal fibroblasts (hDF). wt/wt: wild-type hDF; mut/mut: patient hDF; mut/mut+NSUN3: patient hDF expressing the V5-tagged NSUN3 construct. Lysate of HEK293 cells over-expressing the Flag-tagged NSun3 served as a control. ‘Unspec' indicates a non-specific band detected in human mitochondria by anti-NSun3 antibodies. CBS stands for Coomassie blue staining. (**b**) Oxygen consumption rate in wild-type (wt/wt) and patient-derived cells (mut/mut) as well as in wt/wt and mut/mut cells expressing a V5-tagged NSun3 construct (+NSUN3). Graph is a representative for three independent replicates, *P* values obtained in Student's *t*-test. (**c**) Relative cell growth rate of wt/wt, mut/mut and mut/mut+NSUN3 human dermal fibroblasts in galactose-containing medium normalized by growth rate in glucose-containing medium. (**d**) mtDNA copy-number determination by qPCR, performed in triplicate, of mitochondrial DNA fragments relative to the nuclear *B2M* gene. Statistical analysis was carried out using a two-tailed student's *t*-test. Error bars represent s.d. of the mean. (**e**) qPCR determination of mt-rRNA (12S and 16S) and mt-mRNA (CO1 and CO2) levels compared with GAPDH in wt/wt, mut/mut and mut/mut+NSUN3 cells. qPCR was performed in triplicate and error bars indicate the s.d. of the mean. (**f**) Northern blot analysis of mt-tRNAs in two different wt/wt cells, mut/mut and mut/mut+NSUN3 cells. Cytoplasmic 5S rRNA is used as loading control. (**g**) Mitochondrial *de novo* protein synthesis assessed with ^35^S metabolic labelling. CBS: Coomassie blue stained gel as loading control. (**h**) Quantification of the bands intensities shown in **g** using ImageQuant.

The compound heterozygous *NSUN3* gene variants are predicted to result in a non-functional, truncated protein product (Fig. 1e). Western blot analysis using primary human dermal fibroblasts (hDFs) confirmed the lack of the full-length NSun3 protein in patient-derived cells (Fig. 2a, mut/mut) that could be rescued upon lentiviral transduction with complementary DNA (cDNA) encoding a V5-tagged NSun3 construct (Fig. 2a, mut/mut+NSUN3).

We next asked whether the lack of NSun3 affected mitochondrial function in patient's hDFs. The mitochondrial respiratory chain activity was impaired in the absence of NSun3 (Fig. 2b, Supplementary Note 1). The reduced oxygen consumption rate in NSun3 mutant cells (mut/mut) was rescued by re-expression of exogenous NSun3 cDNA (mut/mut+NSUN3). We further confirmed the compromised respiratory chain performance of hDFs lacking the functional NSun3 protein by growing them in a medium containing galactose as the sole carbon source, therefore forcing the cells to rely more on mitochondrial ATP production. Under these conditions, the patient-derived hDFs (mut/mut) had a lower growth rate than wild-type cells (wt/wt); and this effect was rescued by re-expression of NSun3 (mut/mut+NSUN3; Fig. 2c). These results indicate that NSun3 is necessary for efficient mitochondrial OXPHOS.

We next asked whether the lack of NSun3 affects mitochondrial gene maintenance and expression. The mtDNA copy number was unchanged between wt/wt and mut/mut hDFs (Fig. 2d), consistent with mitochondrial biogenesis being unaffected by the absence of NSun3. We next tested whether the lack of NSun3 affects mtRNA expression by measuring mtRNA levels. In qPCR experiments, we detected an increase in mitochondrial transcript levels (precursor and mature) in mut/mut hDFs that was rescued to normal levels upon *NSUN3* cDNA re-expression (mut/mut+NSUN3) (Fig. 2e). Northern blot analyses confirmed the increase in the levels of mature mt-mRNAs and mt-rRNAs in *NSUN3* mutant cells (Supplementary Fig. 1a). High-resolution northern blot analysis did not reveal any appreciable differences in expression levels of mt-tRNAs in the absence of NSun3 (mut/mut; Fig. 2f), nor did it show any effect on mt-tRNA aminoacylation (Supplementary Fig. 1b). In contrast, measurement of mitochondrial translation by incorporation of radioactively labelled methionine revealed that the mitochondrial *de novo* protein synthesis rate was markedly decreased in the absence of NSun3 (Fig. 2g,h, Supplementary Fig. 1c). Lentiviral transduction of *NSUN3* cDNA resulted in a substantial increase of mitochondrial translation rate in patient's hDF (Fig. 2g,h, Supplementary Fig. 1c). The decrease in mitochondrial protein synthesis rate in mut/mut cells was accompanied with a reduction in the steady-state levels of complex I (Supplementary Fig. 1d,e); complex I is often affected predominately when expression of mtDNA in perturbed^20^. We concluded that in the absence of NSun3 mitochondrial transcripts are upregulated, possibly due to compensatory effects, yet the loss-of-function mutations of NSun3 impair mitochondrial protein synthesis. Thus, altogether these data show that NSun3 is necessary for efficient mitochondrial translation, suggesting that an *NSUN3* defect is responsible for the combined OXPHOS deficiency in the patient.

### NSun3 methylates mitochondrial tRNA^Met^ at position C34

**Figure 3:**
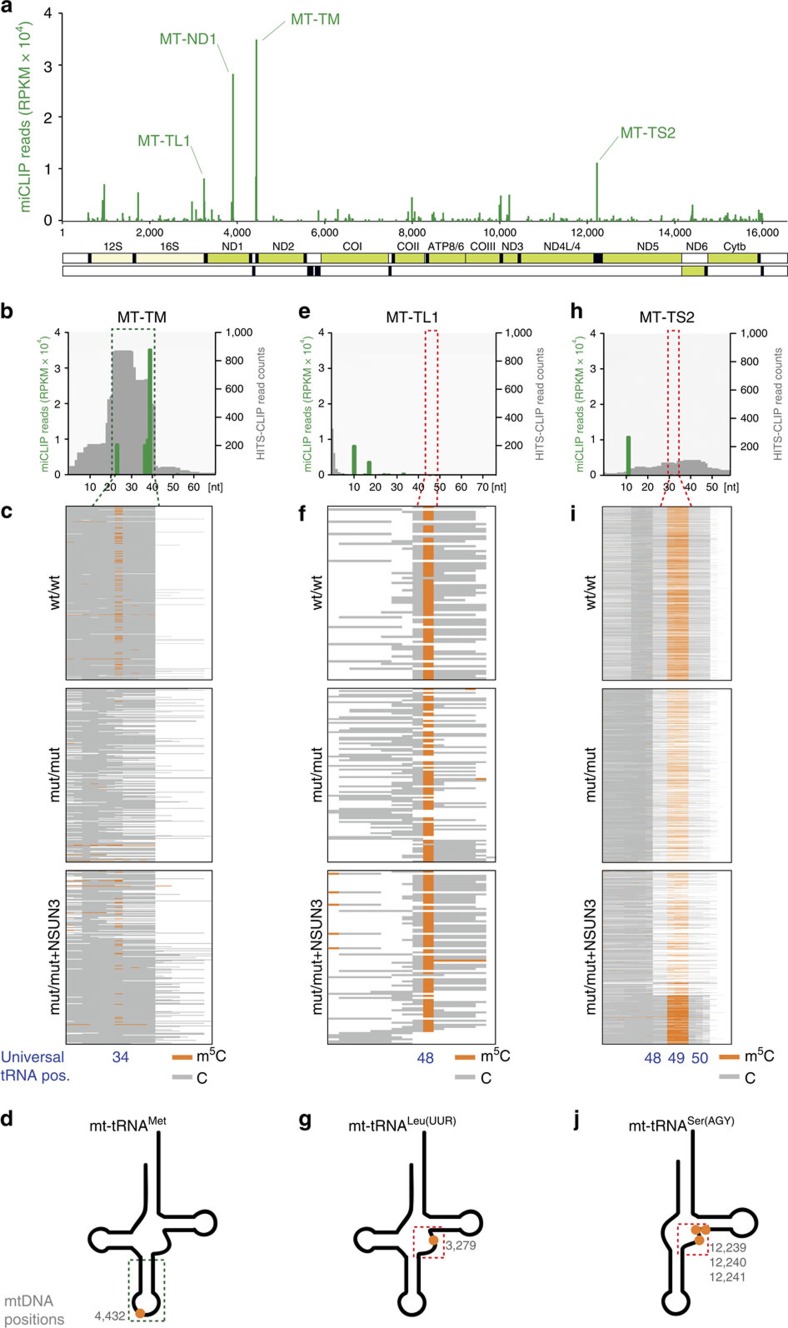
Pathogenic mutations in NSUN3 cause loss of m^5^C34 in mitochondrial tRNA^Met^. (**a**) miCLIP reads mapped to mtDNA (mt-tRNA black, mt-mRNA lime, mt-rRNA yellow). (**b**) miCLIP (green) and HITS-CLIP read counts (grey) corresponding to the *MT-TM* gene coding for mt-tRNA^Met^. The length of mt-tRNA is indicated on the *x* axis. The green dashed line rectangle indicates the position of anticodon arm. (**c**) Methylated (orange) and unmethylated (grey) cytosines (*x* axis) detected by BS RNA-Seq (individual reads on *y* axis) for mt-tRNA^Met^ for wt/wt, mut/mut and mut/mut+NSUN3 cells. (**d**) Schematic structure of mt-tRNA^Met^ and the position of m^5^C (orange circle) in the anticodon arm (green dashed line rectangle). (**e**–**g**) miCLIP and HITS-CLIP reads, BS RNA-Seq and position of m^5^C of MT-TL1/mt-tRNA^Leu(UUR)^ (the details as per **b**–**d**). (**h**–**j**) miCLIP and HITS-CLIP reads, BS RNA-Seq and position of m^5^C of MT-TS2/mt-tRNA^Ser(AGY)^ (details as per **b**–**d**). The red dashed line rectangle (**e**,**h**) and the red dashed line square (**g**,**j**) indicate the tRNA variable region.

NSun3 shows sequence homology to proteins of the NOL1/NOP2/Sun (NSun) domain-containing family (PFAM: 01189) that are class I *S*-adenosylmethionine-dependent methyltransferases (AdoMet-MTase; Supplementary Fig. 2a). Other members of this family have been implicated in post-transcriptional cytosine-5 methylation (m^5^C) of RNA. We recently utilized methylation-individual nucleotide resolution crosslinking and immmunoprecipitation (miCLIP) in a transcriptome-wide approach to identify NSun2-methylated nucleosides^21^. Here we used this method to reveal RNA targets of the related m^5^C RNA methyltransferase NSun3. All RNA:m^5^C methyltransferases contain a catalytic domain with a common structural core and the AdoMet-binding site (Supplementary Fig. 2a). Two conserved active site cysteines, (T**C**_**1**_ and P**C**_**2**_), are required for the transfer of the methyl group (Supplementary Fig. 2a)^22^. Catalytic cysteine (C_1_) forms a covalent bond with the cytosine pyrimidine ring. The second conserved cysteine (C_2_) is required to break the covalent adduct and releases the methylated RNA from the enzyme (Supplementary Fig. 2a,b). Mutating the cysteine C_2_ to alanine (NSun3-C2A) results in the irreversible formation of an RNA–protein complex at the methylation site (Supplementary Fig. 2a–c)^22^,^23^,^24^. Such catalytic intermediate RNA–protein complex can be immunoprecipitated and the attached RNA identified by sequencing. Covalent crosslinking of the catalytically inert protein to the cytosine it tries to methylate, leaves a short peptide at this site and causes the reverse transcriptase to stall^25^, so all sequence reads end around (but not necessarily exactly at) the methylation site^9^,^21^. We performed miCLIP following transfection of the NSun3-C2A catalytic mutant cDNA construct into HEK293 cells (Supplementary Fig. 2d). This analysis showed that, in contrast to NSun2, which mainly methylates cytoplasmic tRNAs^11^,^21^, the vast majority of NSun3 miCLIP detected sites mapped to mtRNAs (Supplementary Fig. 2e,f, Supplementary Data 1), consistent with mitochondrial localization of the protein. Analysis of miCLIP reads revealed a broad spectrum of potential target sites in mtRNA, however, a more substantial enrichment of sequences specific for the following mitochondrial genes was observed: the MT-TM, coding for mt-tRNA^Met^ (26% of all reads mapped to mtDNA and 60% of all mitochondrial tRNA miCLIP loci), MT-ND1, for mitochondrially encoded NADH dehydrogenase 1 (15% of all reads mapped to mtDNA), along with two other mt-tRNAs genes, MT-TL1 (coding for mt-tRNA^Leu(UUR)^) and MT-TS2 (coding for mt-tRNA^Ser(AGY)^) (5% and 4.5% of mtDNA-mapped reads, respectively; Fig. 3a, Supplementary Data 1). Remarkably, miCLIP sequence reads corresponding to the nuclear-encoded, cytoplasmic (ct-) initiator tRNA methionine (ct-tRNA^iMet^) were also enriched (83% of all reads mapped to nDNA; Supplementary Data 1). While these data suggest that NSun3 methylates mitochondrial RNA, they also point towards both the mitochondrial and cytoplasmic tRNA^Met^ as key substrates for the enzyme.

To provide further insights into NSun3 mtRNA targets, we additionally performed ultraviolet crosslinking and immunoprecipitation coupled with high-throughput sequencing (HITS-CLIP) using mitochondrial lysates of HEK293T cells. To compensate for a possible bias caused by antibody–epitope interactions, we used a NSun3-Flag construct in the HITS-CLIP assay (rather than c-myc in the miCLIP approach). This analysis revealed that, among all mitochondrial transcripts, only MT-TM-specific reads had a significantly enriched read count (Supplementary Data 2) compared with a control. As HITS-CLIP is able to map RNA-protein binding footprint regions at a resolution of ∼30 to 60 nucleotides^26^, we looked at the exact positions of MT-TM-specific reads. This analysis revealed that NSun3 targets predominantly the mt-tRNA^Met^ anticodon loop (Fig. 3b, Supplementary Fig. 2g–i).

We applied a third technique to determine which specific cytidine residues are methylated by NSun3 and we examined the differences in RNA m^5^C patterns on *NSUN3* loss-of-function. To this end, we performed high-throughput sequencing of cDNA libraries obtained after bisulfite treatment of RNA of wild-type hDFs (wt/wt), patient hDFs lacking the functional NSun3 protein (mut/mut) and mut/mut hDFs expressing a *NSUN3* cDNA (mut/mut+NSUN3). The bisulfite RNA sequencing (BS RNA-Seq) analysis revealed that methylation in mt-tRNA^Met^ (MT-TM) was decreased to background levels in *NSUN3* mutant cells (mut/mut), while other top mtRNAs with sites enriched by miCLIP (Fig. 3a; MT-TL1, MT-TS2, MT-ND1) showed no changes in the level of cytosine-5 methylation in *NSUN3* mutant cells (mut/mut), when compared with wt/wt or the cells transduced with exogenous *NSUN3* (mut/mut+NSUN3; Fig. 3b–i; Supplementary Fig. 3a,b; Supplementary Data 3). We did not detect m^5^Cs in the MT-ND1 transcript in any of the cell lines examined (Supplementary Data 3, Supplementary Fig. 4a,b), suggestive for non-specific miCLIP hit for this gene, possibly owing to the structural features of the identified MT-ND1 RNA region (Supplementary Fig. 4c), in combination with sub-optimal expression levels of NSun3 in the miCLIP experiment. The m^5^C detected in mt-RNA^Met^ occurred at universal tRNA position 34 (‘wobble' base) in the anticodon loop (corresponding to mtDNA position 4432) and overlapped with the miCLIP and HITS-CLIP-detected sites in this mt-tRNA (Fig. 3b–d). Although, we detected m^5^C in mt-tRNA^Leu(UUR)^ (MT-TL1) and mt-tRNA^Ser(AGY)^ (MT-TS2), we did not uncover any appreciable differences in this modification between wt/wt, mut/mut or mut/mut+NSUN3 hDFs (Fig. 3f,g,i,j) and the mt-tRNA methylation positions did not overlap with miCLIP sites (Fig. 3e,h). The bisulfite RNA sequencing also revealed m^5^C in other mt-tRNAs, namely mt-tRNA^His^, mt-tRNA^Glu^, mt-tRNA^Phe^ (Supplementary Fig. 3c–h). However, none of these other methylated mt-tRNAs exhibited reduction in the m^5^C levels in the patient fibroblasts (Supplementary Fig. 3c–h). We further analysed the BS RNA-Seq reads to determine whether the ct-tRNA^iMet^, highly enriched in the miCLIP experiments (Supplementary Data 1), is also a target for NSun3. We detected m^5^C at position 48 of ct-tRNA^iMet^ (Supplementary Fig. 4d,e), however, we found no changes in the m^5^C levels in the patient fibroblasts (Supplementary Fig. 4d). This result is consistent with the previously published data reporting m^5^C48 in ct-tRNAi^Met^ being introduced by NSun2 (ref. 11). We deduced that miCLIP enrichment of ct-tRNA^iMet^ by NSun3 is likely to be caused by close sequence and structure similarity of the anticodon loop of mt-tRNA^Met^ and ct-tRNA^iMet^ (Supplementary Fig. 4f,g). In the miCLIP experiment, overexpressed NSun3-C2A is released from mitochondria on cell lysis, which allows for interaction with ct-tRNA^iMet^ and a subsequent pull-down. This interaction is prevented *in vivo* by stringent regulation of NSun3 compartmentalization. Collectively, the combined approach of miCLIP, HITS-CLIP and BS RNA-Seq confirm that cytosine-5 methylation of mt-tRNA^Met^ at position 34 is mediated by NSun3.

### Methylation of mt-tRNA^Met^ is necessary for f^5^C formation

**Figure 4:**
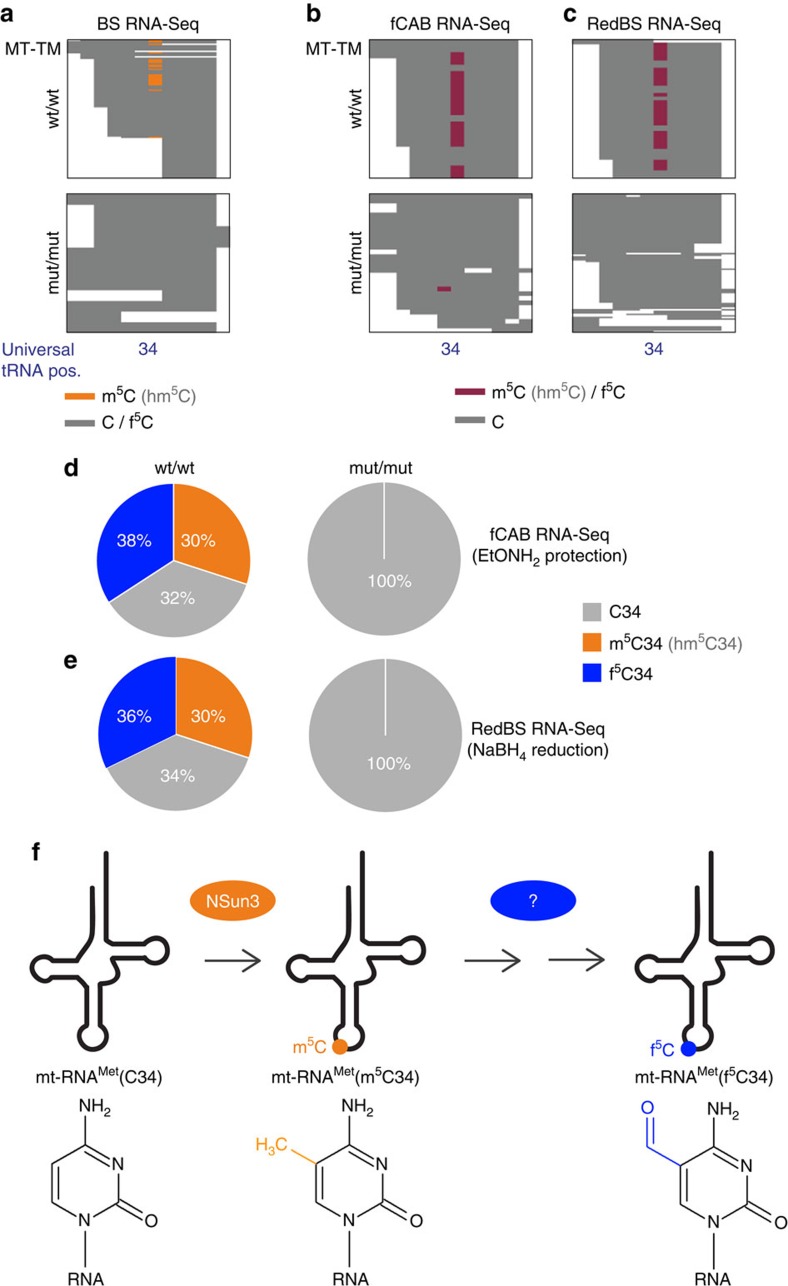
m^5^C34 in mt-tRNA^Met^ is a necessary precursor for f^5^C34. (**a**) Heatmaps of BS RNA-Seq reads for mt-tRNA^Met^ (MT-TM) for wt/wt and mut/mut cells, showing cytosines of individual reads (on *y* axis). Methylated (and hydroxymethylated) cytosines are shown in orange, while unmodified or formylated cytosines are shown in grey (*x* axis). (**b**) Heatmaps of fCAB RNA-Seq reads for mt-tRNA^Met^ (MT-TM) for wt/wt and mut/mut cells, showing cytosines of individual reads (on *y* axis). Purple indicates methylated, hydroxymethylated or formylated, while unmodified cytosines are shown in grey. (**c**) Heatmaps of RedBS RNA-Seq for mt-tRNA^Met^ (MT-TM) for wt/wt and mut/mut cells showing cytosines of individual reads (on *y* axis). Purple indicates methylated, hydroxymethylated or formylated, while unmodified cytosines are shown in grey. (**d**) Summary of the fCAB RNA-Seq results for wt/wt and mut/mut cells. (**e**) Summary of the RedBS RNA-Seq results for wt/wt and mut/mut cells. (**f**) Graphical overview of the mt-tRNA^Met^ C34 formylation pathway. NSun3 methylates unmodified C34 into m^5^C34, which is then further processed (responsible enzyme(s) unknown) into f^5^C34.

Although never shown in human mt-tRNA, several studies have demonstrated the presence of f^5^C at position C34 of mature mammalian mt-tRNA^Met^ (refs 27, 28). Since bisulfite treatment does not distinguish between f^5^C and unmodified cytosines^29^ (Fig. 4a), we applied two high-throughput techniques to detect f^5^C in mitochondrial RNA from wt/wt and mut/mut samples. First, reduced bisulfite sequencing (RedBS-Seq) relies on the chemical reduction of f^5^C to hm^5^C by NaBH_4_; hm^5^C is then detected in the same manner as m^5^C (ref. 29). Second, *O*-ethylhydroxylamine was used to protect f^5^C against conversion in f^5^C chemically assisted bisulfite sequencing (fCAB-Seq)^30^. Consistent with the previously published data for other mammalian species, both techniques detected f^5^C34 in mt-RNA^Met^ in the wt/wt sample (36 and 38% for RedBS-Seq and fCAB-seq, respectively). However, no f^5^C was detected in mut/mut samples using either of the methods (Fig. 4b–e). We concluded that methylation of position C34 by NSun3 is necessary for f^5^C34 formation in mt-tRNA^Met^ (Fig. 4f).

## Discussion

Here we have identified the mitochondrial substrate for the previously uncharacterized cytosine-5 RNA methyltransferase NSun3, found mutated in a patient with mitochondrial disease. The NSun3-null patient fibroblasts have a striking deficiency of m^5^C and f^5^C at position 34 (‘wobble' base) in the anticodon of mt-tRNA^Met^, consistent with a precursor–product relationship for these two modifications. We also show that this deficiency is associated with impaired mitochondrial translation and OXPHOS.

The presence of m^5^C at position 34 has not been reported previously in mammalian mt-tRNA^Met^ (ref. 31). However, this position has been known to contain 5-formylcytidine (f^5^C). Although f^5^C has never been formally reported in human, this modification has been considered as universal and detected in a number of other species including cow, rat, chicken, frog, squid, fruit fly and nematode^27^,^28^. Therefore, our finding of m^5^C34 modification in mt-tRNA^Met^ as a key target for NSun3 might be considered unexpected, as it would be mutually exclusive with the previously reported f^5^C34 (cytidine carbon 5 is modified in both cases). Despite the widespread occurrence of f^5^C34 in mt-tRNA^Met^, the enzymatic activity responsible for its formation is unknown. However, a recent *in vivo* isotope-tracing experiment in a mouse model has revealed that m^5^C in RNA can be oxidatively metabolized first to 5-hydroxymethylcytosine (hm^5^C) and subsequently to f^5^C (ref. 32). With this in mind, we analysed f^5^C content in mt-tRNA^Met^ in NSun3-null and control cells. We found that, although f^5^C34 is indeed present in mt-tRNA^Met^ in wild-type human mitochondria, no f^5^C was detected in NSun3-null patient samples. The proportion of mt-tRNA^Met^ that contain f^5^C34 in human dermal fibroblasts has not been determined previously. Our study revealed that ∼36 to 38% of the mt-tRNA^Met^-specific RNA-Seq reads contain f^5^C34 in wt/wt cells. This proportion might be biased by incomplete reduction of f^5^C to hm^5^C by NaBH_4_ or not complete protection of f^5^C by *O*-ethylhydroxylamine, therefore the actual percentage of f^5^C34 in the mature mt-tRNA^Met^ might be higher. Nonetheless, the complete lack of f^5^C in the NSun3-null cells is consistent with methylation of C34 by NSun3 being necessary for formation of f^5^C34 in mt-tRNA^Met^ (Fig. 4f).

The dynamics and oxidative metabolism of C5-methylation has recently been the subject of intense investigation in DNA. The 10–11 translocation (Tet) family of dioxygenases mediates the sequential removal of m^5^dC through an oxidative pathway involving the intermediates hm^5^dC and f^5^dC (ref. 33). Further studies have demonstrated that f^5^dC can be a stable DNA modification, suggesting that it has its own unique biological function in addition to being an intermediate in m^5^dC removal^34^. Tet-mediated oxidation of m^5^C to hm^5^C and f^5^C in RNA has been shown *in vitro*, although much less efficient than the corresponding oxidation of m^5^dC in DNA^35^. Further studies will be necessary to identify a dioxygenase mediating m^5^C to f^5^C conversion in human mitochondria and to establish its regulatory mechanisms (Fig. 4f).

Why has m^5^C34 in mt-tRNA^Met^ not been detected in previous studies, despite relatively high detection levels in BS RNA-Seq? In the reports on post-transcriptional modifications of mt-tRNA^Met^ only mature mt-tRNA species were analysed. In contrast, our miCLIP and BS RNA-Seq approaches are able to detect m^5^C both in precursor and mature human mt-tRNA^Met^. We consider that m^5^C could be introduced early in the mtRNA maturation pathway, so that predominately precursor mtRNA contains this modification. This is in line with other published work, where f^5^C34 has been detected in addition to m^5^C34 in mammalian ct-tRNAs^Leu(CAA)^. In this case, another member of the NOL1/NOP2/Sun family, NSun2, is responsible for catalysing ct-tRNAs^Leu(CAA)^ m^5^C34 with a preference for precursor tRNA^11^,^12^,^36^. Furthermore, the BS RNA-Seq method used is unable to distinguish between m^5^C and its oxidized derivative hm^5^C, therefore the NSun3-mediated methylation consistently detected at ∼30% by BS RNA-Seq of wt/wt cells (Fig. 3c) might be overestimated.

How does the lack of NSun3-mediated mt-tRNA^Met^ C34 methylation affect mitochondrial translation? Mitochondrial protein synthesis displays unique features as compared with its cytoplasmic counterpart. In human mitochondrial mRNAs, methionine is encoded either by the universal AUG or the unconventional AUA (isoleucine in the universal genetic code). Also, mitochondrial translation in many organisms, including humans, occasionally initiates with methionine encoded by the universal isoleucine codons AUU or AUC. There is only one mitochondrial tRNA^Met^ that decodes all of these AUN codons^27^,^28^. It has been shown *in vitro* that f^5^C34 in human mt-RNA^Met^ allows for decoding of AUG and the non-traditional codons AUA, AUU and AUC^28^,^37^. Our data on methylation by NSun3 providing a necessary precursor for f^5^C34 in mt-RNA^Met^, as well as the general mitochondrial translation defect observed in patient-derived NSun3-null cells, are compatible with the existing evidence on less efficient decoding by unmodified mt-RNA^Met^.

Our study also constitutes a valuable resource of human mt-tRNA modifications as we provide previously unreported modifications. We detected eight cytosine-5 methylations in six mitochondrial tRNAs. We confirm the presence of five cytosine methylations that were described previously in human and bovine mt-tRNA: m^5^C49 of mt-tRNA^Glu^, m^5^C48 of mt-tRNA^Leu(UUR)^ and m^5^C48, -49, -50 of mt-tRNA^Ser(AGY)^ (refs 31, 38, 39). Apart from m^5^C34 of mt-RNA^Met^, we also detect the presence of two m^5^C modifications in human mt-tRNA that have not been reported previously: m^5^C47 of mt-tRNA^His^ and m^5^C47 mt-tRNA^Phe^, both occurring in the variable region of the tRNAs (Supplementary Table 1).

In summary, we have identified NSun3 as a novel human m^5^C RNA methyltransferase and show its target specificity, mitochondrial tRNA^Met^. Mutations in *NSUN3*, found in a patient with mitochondrial disease, were associated with hypomethylation and the absence of formylation of cytosine residue at position 34 of mitochondrial tRNA^Met^ leading to impaired mitochondrial translation. This is highly suggestive of a causal link between mutations in *NSUN3* and mitochondrial disease. Our study highlights the importance of epitranscriptome modification for optimized mitochondrial protein translation and faithful cellular function, highlighting a novel role for m^5^C as precursor of f^5^C in human mitochondria.

*Note added in proof:* After we submitted this report, similar conclusions on the role of NSUN3 were reported (Nakano *et al*. *Nat. Chem. Biol*. 2016 May 23. doi:10.1038/nchembio.2099).

## Methods

### Detection of NSUN3 variants

Whole-exome sequencing was performed using a SureSelect Human All Exon 50 Mb Kit (Agilent) for enrichment and a HiSeq2500 (Illumina) for sequencing^18^ Read alignment to the human genome (UCSC Genome Browser build hg19) was done with Burrows–Wheeler Aligner (BWA, v.0.7.5a) and single-nucleotide variants and small insertions and deletions were detected with BWA (v.0.7.5a) and SAMtools (version 0.1.19). Given that mitochondrial disorders are rare, we excluded variants with a frequency >0.1% in 7,000 control exomes and public databases. On the basis of a recessive model of inheritance, we next searched for genes carrying predicted homozygous or compound heterozygous nonsynonymous variants followed by a filter for genes encoding proteins with a predicted mitochondrial localization^40^. Written informed consent of diagnostic and research studies was obtained from all individuals investigated or their guardians, and the ethics committee of the Technische Universität München approved the study.

### NSun3 cDNA constructs

Full-length cDNA constructs for NSun3 in the pCMV6-Entry-Myc vector were obtained from OriGene. Site-directed mutagenesis to generate the miCLIP-mutant was performed using the QuikChange II Site-Directed Mutagenesis Kit from Agilent as per the manufacturer's instructions (Supplementary Table 2). Overexpression of NSun3 in mut/mut human fibroblasts was performed through lentiviral gene transfer using the ViraPower HiPerform Lentiviral TOPO Expression Kit (Life Technologies). The full-length NSun3 cDNA was obtained from DNASU Plasmid Repository (HsCD00439330) and was cloned into the pLenti6.3/V5-TOPO vector system (Thermo Fisher) (Supplementary Table 2). For NSun3 overexpression in HeLa cells, full-length cDNA was cloned into a plasmid encoding FLAG.STREP2-tag and the resulting fragment was cloned into pcDNA5-FST2 as previously described^41^ (Supplementary Table 2).

### Cell culture and transfection

HEK293 cells, HeLa cells and primary hDFs were grown in DMEM (Life Technologies) supplemented with 10% fetal bovine serum at 37 °C in a humidified atmosphere with 5% CO2. HEK293 cells were transfected with NSun3 wild-type or NSun3 miCLIP-mutant constructs using Lipofectamine 2000 (Life Technologies) and collected 24 h later. For immunofluorescence studies, HeLa cells were transfected with the NSun3 FLAG.STREP2-tagged construct using Lipofectamine 2000 and were analysed 48 h post transfection.

Lentiviral particles containing the packaged pLenti NSun3 and NSun3-V5 expression constructs were generated by cotransfecting HEK 293FT cells with the packaging plasmid mix and the NSun3 or NSun3-V5 expression construct, respectively using Lipofectamine 2000. Twenty four hours after transfection the medium containing the DNA- Lipofectamine 2000 mix was replaced with high glucose DMEM supplemented with 10% FBS and the cells were incubated for another 48 h. The virus-containing supernatant was collected and used for subsequent transduction of control and patient fibroblasts. Stably transduced fibroblasts were selected using Blasticidin (Thermo Fisher) for at least 2 weeks and kept under selection for any further analysis.

Flp-In T-Rex HEK293T cells were maintained in DMEM supplemented with 10% tetracycline-free fetal bovine serum (Biochrom AG), 100 μg ml^−1^ Zeocin and 15 μg ml^−1^ Blasticidin (Life Technologies). Cells were stably transfected with the NSun3 FLAG.STREP2-tagged construct with Lipofectamine 2000. After 1 day, medium with selective antibiotics (50 mg ml^−1^ hygromycin and 15 mg ml^−1^ blasticidin (Life Technologies) was used to select cells with the stably integrated transgene.

For cell proliferation measurements cells were plated at a density of 50,000 fibroblasts per well of a six-well plate and confluency was measured every 6 h using an IncuCyte ZOOM live-cell imaging system (Essen BioScience).

### Respiratory chain assay

Fibroblasts were seeded at a density of 20,000 cells per well in 80 μl of culture media in a XF 96-well cell culture microplate (Seahorse Bioscience) and incubated overnight at 37 °C in 5% CO_2_. Culture medium was replaced with 180 μl of bicarbonate-free DMEM and cells were incubated at 37 °C for 30 min prior measurement. Oxygen consumption rate was measured using a XF96 Extracellular Flux Analyser (Seahorse Biosciences). Oxygen consumption rate was determined with no additions; after addition of oligomycin (1 μM); after addition of carbonyl cyanide 4-(trifluoromethoxy) phenylhydrazone (FCCP, 0,4 μM); and after addition of rotenone (2 μM) (additives purchased from Sigma at highest quality).

### Immunodetection of proteins

Immunofluorescence analysis and cell fractionation were performed essentially as described previously^42^. In brief, HeLa cells were transiently transfected to express NSun3-FLAG-Strep2 for 2 days. Cells were fixed with 4% formaldehyde for 15 min and permeabilized with 1% TritonX for 5 min before blocking with 10% fetal bovine serum for 1 h. The following primary antibodies were used: mouse anti-FLAG IgG (Sigma F3165, 1/200) and rabbit anti-TOM20 (Santa Cruz Biotechnology sc-11415, 1/500). Secondary antibodies were Alexa Fluor 488 goat anti-rabbit and Alexa Fluor 594 goat anti-mouse (both Life Technologies, 1/1000). Next, cells were mounted with DAPI-containing ProLong Gold Antifade (Life Technologies) and immunofluorescence images were captured using a Zeiss Observer microscope and Apotome system.

For immunoblot analysis, equal amounts of proteins corresponding to total cell lysates or protein fractions were subjected to SDS–PAGE electrophoresis, semi-dry transferred to nitrocellulose membranes, blocked in 5% non-fat milk (Marvel) in PBS for 1 h and incubated with specific primary antibodies in 5% non-fat milk in PBS for 2 h or overnight. The blots were further incubated with HRP-conjugated goat anti-rabbit and anti-mouse (Promega W401B and W4028, 1/2,000) for 1 h and visualized using ECL (GE Healthcare). The following primary antibodies were used: NSun3 (Sigma Aldrich HPA036181, 1/250), TOM22 (Abcam ab10436, 1/2,000), GAPDH (Abcam ab9482, 1/2,000), mt-SSB1 (kindly donated by Prof. D. Kang, 1/4,000), Histone H3 (Abcam ab1791, 1/4,000), beta actin (Sigma A1978, 1/75,000) and Total OXPHOS Human WB Antibody Cocktail (Abcam ab110411, 1/1,000).

### Mitochondrial DNA copy number and deletion analysis

Total cellular DNA was isolated from fibroblasts using a DNeasy Blood and Tissue kit (Qiagen) according to the manufacturer's protocol. Copy numbers of mtDNA were determined by quantitative PCR^43^. All primers can be found in Supplementary Table 2.

### Mitochondrial transcript analysis

Total RNA from fibroblasts was extracted using TRIzol and treated with Turbo DNase according to the manufacturer's protocol. For PCR, 2 μg of DNase treated RNA was reverse transcribed using Omniscript RT kit (Qiagen) with 0.5 μM random hexamers and 0.5 μM oligo dT primers. Primer sequences can be found in Supplementary Table 2. For northern blot analysis, total RNA was resolved on 1% agarose gels containing 0.7 M formaldehyde in 1 × MOPS buffer (mt-mRNAs) or on 5% UREA–PAGE (mt-tRNAs), transferred to a nylon membrane in 2 × SSC, ultraviolet-crosslinked to the membrane and hybridized with radioactively labelled PCR fragments corresponding to appropriate regions of mtDNA (mt-mRNAs) or T7-transcribed radioactive RNA-probes (mt-tRNAs). Mt-tRNA aminoacylation analysis was performed as previously described^44^. Briefly, total RNA was resuspended in 10 mM NaOAc (pH 5.0) and kept at 4 °C to preserve the aminoacylation state. For deacylated control, the pellet was resuspended in 200 mM Tris-HCl (pH 9.5) and incubated for 5 min at 75 °C, followed by RNA precipitation and resuspension in 10 mM NaOAc (pH 5.0). Next, 15 μg of RNA was separated on a 6.5% polyacrylamide gel (19:1 acrylamide:bisacrylamide) containing 8 M urea in 0.1 M NaOAc (pH 5.0) at 4 °C and electroblotted (Bio-Rad, Trans-Blot Cell) onto a nylon transfer membrane (Hybond, GE). Following ultraviolet crosslinking, the membrane was hybridized with appropriate radiolabelled riboprobes, washed and imaged using a PhosphorImager.

### ^35^S-methionine metabolic labelling of mitochondrial proteins

Metabolic labelling was performed essentially as described previously (ref. 45). In brief, fibroblasts were grown as described above until at 80% confluency, then medium was replaced with methionine/cysteine-free DMEM (Sigma) supplemented with 2 mM L-glutamine, 48 μg ml^−1^ cysteine and 50 μg ml^−1^ uridine for 2 × 10 min, followed by methionine/cysteine-free DMEM medium containing 10% (v/v) dialysed FCS and emetine dihydrochloride (100 μg ml^−1^) to inhibit cytosolic translation for 10 min before addition of 120 μCi ml^−1^ of [^35^S]-methionine. Labelling was performed for 15 min. SDS–PAGE was performed on 30 μg of protein and products were imaged using a PhosphorImager.

### Methylation-iCLIP (miCLIP)

For miCLIP experiments, collected cells were lysed in lysis buffer consisting of 50 mM Tris-HCL pH 7.4, 100 mM NaCl, 1% NP-40, 0.1% SDS, 0.5% sodium deoxycholate. Lysates were then treated with high concentration of DNase and low concentration of RNaseI to partially fragment RNAs. Lysates were cleared by centrifugation at 13,000 r.p.m. for 15 min at 40 °C and then incubated with Protein G Dynabeads (Life Technologies) in the presence of an anti-Myc antibody (9E10, Sigma). Following stringent washing, 3′-end dephosphorylation was performed with T4 PNK (New England Biolabs) before addition of a preadenylated linker using RNA ligase (New England Biolabs). 5′-end labelling was then performed using T4 PNK and ^32^P-ATP before protein–RNA complexes were eluted and run on denaturing gels. Next, nitrocellulose transfer was performed and the radioactive signal was used to dissect nitrocellulose pieces that contained NSun2-partially digested RNA complexes. RNA was recovered by incubating the nitrocellulose pieces in a buffer containing Proteinase K and 3.5 M urea. Reverse transcription was performed using oligonucleotides containing two inversely oriented adaptor regions separated by a BamHI restriction site. cDNAs were size-purified on TBE-Urea gels before being circularized by CircLigase II (Epicentre). Circularized cDNAs were then annealed to an oligonucleotide complementary to the BamHI site and then BamHI digested. Linearized cDNAs were then PCR-amplified using primers complementary to the adaptor regions using 25 cycles of PCR. Libraries were then subjected to high-throughput sequencing using the Illumina HiSeq2000 platform. The details of the constructs used in the miCLIP experiments are provided in Supplementary Table 2.

### Processing and mapping of miCLIP reads

To reduce amplification bias, the primers used for reverse transcription during miCLIP experiments were designed to include a six-nucleotide random barcode at positions 1–3 and 8–10 to enable tracing of individual cDNAs. Reads were demultiplexed using the experimental barcode at positions 4–7, and reads with identical random barcodes, representing PCR products, were filtered. The number of different random barcodes for each unique read, which represented cDNA counts, was stored for further analysis. Barcodes were trimmed from the 5′-end, and the adaptor sequence ‘5′- AGATCGGAAGAGCGGTTCAG -3′' from the 3′-end of the reads with cutadapt (ref. 46; options: ‘-O 4 –e 0.06'), and only reads with a minimal length of 18 nucleotides (nt) were retained.

Trimmed miCLIP reads were mapped to the human reference genome (UCSC GRCh37/hg19) by using bowtie (ref. 47) with parameters *‘–m 1 –v 1 –best –strata*' to select uniquely mapping reads allowing one mismatch. Our initial analysis showed that miCLIP reads truncated predominantly at single cytosines. Methylation sites were thus inferred from miCLIP read truncation positions by assigning the read counts to the closest cytosine within ±2 nt of the truncation site. Pooled read counts per cytosine were normalized per million uniquely mapping reads (RPM). If not stated otherwise, only high-confidence methylation sites with normalized read counts>50 RPM in at least two out of three replicates were selected for downstream analyses. Transcript annotations were performed based on ENSEMBL annotations (ENSEMBL 74).

### RNA bisulfite sequencing (BS RNA-Seq)

After enrichment for mitochondria by differential centrifugation in homogenization buffer (10 mM Tris-HCl (pH 7.4), 0.6 M mannitol, 1 mM EDTA), RNA was extracted from NSun3 patient primary fibroblasts and wild-type controls and DNase and Ribo-zero gold (Illumina) treated. Bisulfite conversion of the remaining RNA fraction was performed using the Imprint DNA Modification kit (Sigma). The reaction mixture was incubated for three cycles of 5 min at 90 °C followed by 1 h at 60 °C and then desalted with Micro Bio-spin 6 chromatography columns (Bio-Rad). RNA was desulphonated by adding an equal volume of 1 M Tris (pH 9.0) and incubated for 1 h at 37 °C, followed by ethanol precipitation. About 120 ng of bisulfite-converted RNA was end repaired with T4 PNK (New England Biolabs) and used for library generation, following the manufacturer's protocol (TruSeq Small RNA library preparation kit (Illumina)). Quality and concentration was assessed with a D1000 Screentape for TapeStation (Agilent). Libraries were subjected to high-throughput sequencing using the Illumina MiSeq/HiSeq platform.

### fCAB RNA-Seq and RedBS RNA-Seq

For both techniques, 1 μg of mitochondrial enriched, DNase and Ribo Zero treated RNA of patient and wild-type fibroblasts was used as starting material. BS RNA-Seq was performed in parallel as a control. Chemical reduction of f^5^C in RedBS RNA-Seq was accomplished by adding 20 μl freshly prepared NaBH_4_ (40 mM). After 15 min incubation at room temperature, another 20 μl NaBH_4_ was added for a further 15 min (ref. 29). To protect f^5^C against bisulfite conversion in fCAB RNA-Seq, RNA was incubated with 10 mM *O*-ethylhydroxylamine for 2 h at 37 °C (ref. 30). Further bisulfite treatment and library preparation was similar to BS RNA-Seq.

### UV crosslinking and immunoprecipitation (HITS-CLIP)

A covalent bond between NSun3 protein and RNA in HEK293T cells expressing NSun3.FLAG.STREP2 was induced by irradiation with ultraviolet (UV) light (250 mJ cm^−2^). After cell collection, mitochondria were extracted by differential centrifugation. Pelleted mitochondria were lysed and RNA was partially digested with 4 μl micrococcal nuclease (NEB) for 15 min at room temperature in the presence of 3 μl TURBO DNase. The reaction was stopped by adding 12 μl SUPERasin (ThermoFisher Scientific). After immunoprecipitation with FLAG M2 affinity beads (Sigma) and elution with FLAG peptide as recommended by the manufacturer, proteins were digested with 4 mg ml^−1^ Proteinase K. RNA was end repaired with Antarctic Phosphatase and T4 Polynucleotide Kinase. Library preparation and high-throughput sequencing were similar as for BS RNA-Seq.

### Processing and Mapping of BS RNA-Seq reads

BS RNA-Seq reads were quality trimmed, adaptors were removed and the reads were mapped to the computationally bisulfite-converted human reference genomes (UCSC GRCh37/hg19) with Bismark (ref. 48; parameters: human: *–non-directional –n 1 –l 40*). Methylation levels for all cytosines with a coverage of>5 reads (5 × ) were inferred with the Bismark methylation_extractor tool. Methylation levels were compiled with and without removing potential PCR duplicates to account for tRNA fragments shorter than read length.

### Processing and mapping of HITS-CLIP data

Quality trimming and 3′-end adaptor clipping of sequenced reads were performed simultaneously with Trim galore! ( http://www.bioinformatics.babraham.ac.uk/projects/trim_galore) Reads longer than 20 nt were aligned to the human reference genome (hg19 with mtDNA replaced with rCRS) with Bowtie2 (ref. 49). After counting with Rsubread^50^ and normalization, reads from NSun3 overexpression samples were compared with reads of an unrelated protein (ENSG00000182362) with EdgeR (ExactTest with dispersion 0.1)^51^.

### Processing and mapping of fCAB RNA-Seq and RedBS RNA-Seq data

BS RNA-Seq, fCab RNA-Seq and RedBS RNA-Seq data were pre-processed (both quality trimming and adaptor removal) with Trim Galore! Reads longer than 20 nt were mapped to the computationally bisulfite-converted human mitochondrial genome (NC_012920) with Bismark^49^.

### Data availbility

Source data are provided in Supplementary Fig. 5 and Supplementary Fig. 6. The accession number sequencing data is available from the Gene expression Omnibus (GEO) with accession number GSE66012. All sequencing data related to the patient fibroblasts is available at the European Genome-phenome Archive with accession code EGAS00001000164. The authors declare that the data supporting the findings of this study are available within the article and its Supplementary Information files.

## Additional information

**How to cite this article**: Van Haute, L. *et al*. Deficient methylation and formylation of mt-tRNA^Met^ wobble cytosine in a patient carrying mutations in NSUN3. *Nat. Commun.* 7:12039 doi: 10.1038/ncomms12039 (2016).

## Supplementary Material

Supplementary Information Supplementary Figures 1-6, Supplementary Tables 1-2, Supplementary Note 1 and Supplementary References

Supplementary Data 1 Summary of NSUN3 miCLIP sites

Supplementary Data 2 Summary of NSun3 HIST-CLIP results

Supplementary Data 3 NSUN3 miCLIP sites (+/- 5 nucleotides) covered by RNA BS seq in hDF
