# Supplementary material for: Deficient methylation and formylation of mt-tRNAMet wobble cytosine in a patient carrying mutations in NSUN3
Source: Nat Commun. 2016 Jun 30;7:12039. doi: 10.1038/ncomms12039 (PMC4931328; doi:10.1038/ncomms12039)
Supplement: Supplementary Information — Supplementary Figures 1-6, Supplementary Tables 1-2, Supplementary Note 1 and Supplementary References [file ncomms12039-s1.pdf]

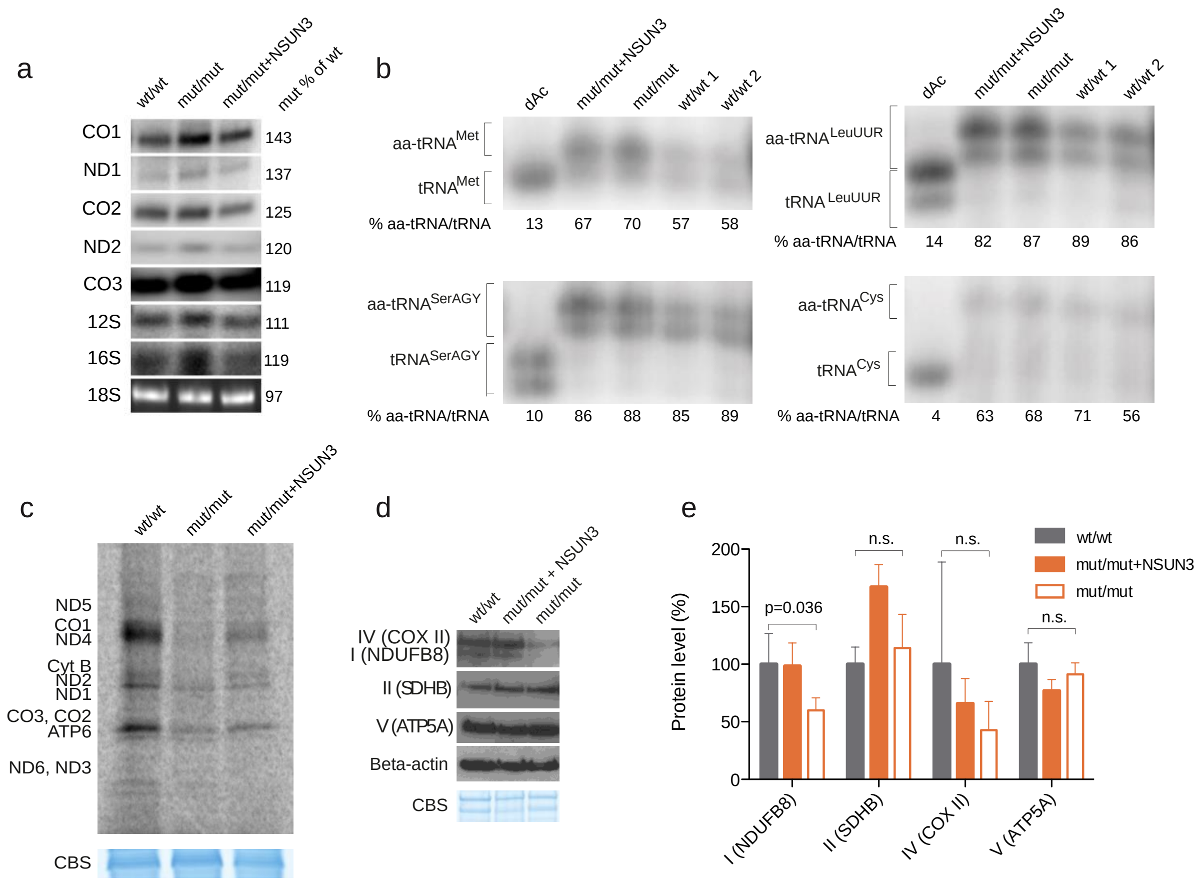

Supplementary Fig. 1:
(a) Northern blot analysis of mature mt-mRNA. 18S is used as loading control.
(b) Analysis of aminoacyl tRNAs by northern blotting and the percentage of aminoacylated tRNA relative to nonaminoacylated tRNA.
(c) Additional ^{35}S metabolic labelling to assess mitochondrial *de novo* protein translation.
(d) Representative example of western blot analysis of NDUFB8, SDHB, COX II, and ATP5A for wt/wt, mut/mut + NSUN3 and mut/mut, cells. CBS: Coomassie blue staining.
(e) Quantification of 3 western blot experiments for NDUFB8, SDHB, COX II, and ATP5A for wt/wt, mut/mut + NSUN3 and mut/mut cells. Data were statistically analysed by two-tailed Student's t-Test. Error bars represent standard deviation of the mean.

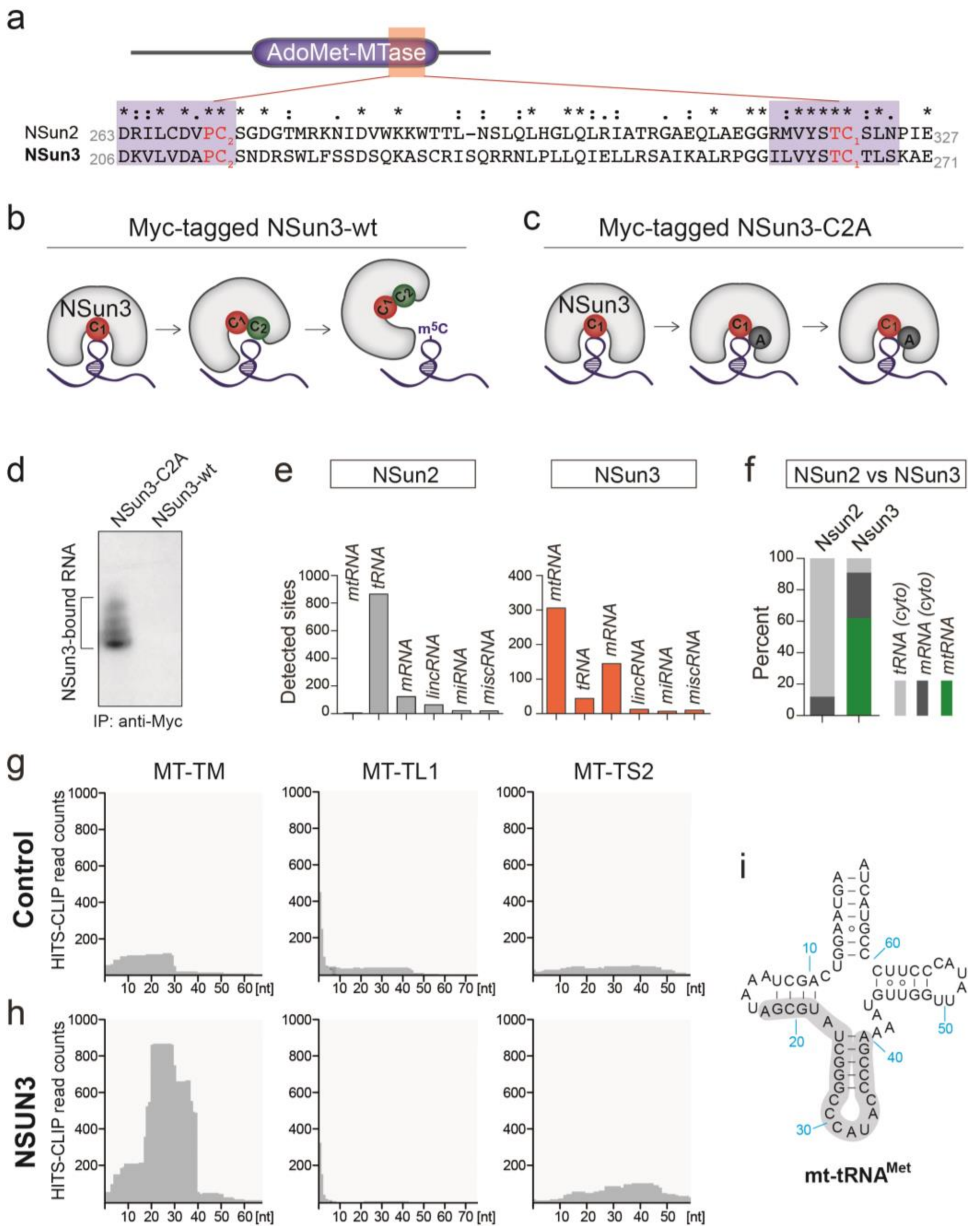

Supplementary Fig. 2:

(a) Sequence alignment of S-adenosylmethionine-dependent methyltransferase domain (AdoMet-MTase) of NSun2, and 3. Red letters indicate the catalytic active sites. Cysteine (C_2) is mutated to generate the miCLIP constructs.

(b-c) Schematic representation of the NSun3-mediated formation of m5C (b) or the covalent bond at the methylated site in the C2 mutant (NSun3-C2A) (c).

(d) RNA released after NSun3 immunoprecipitation and RNase treatment resolved in a polyacrylamide gel. C2A: construct carrying the point C2>A mutation; wt: wild-type construct.

(e) Number of sites in mitochondrial RNA, nuclear-encoded tRNA, mRNA, long intervening noncoding RNAs (lincRNA), miRNA, miscellaneous RNA (miscRNA) targeted by NSun2 (left hand panel) or NSun3 (right hand panel).

(f) Percentage of sites in nuclear-encoded tRNA and mRNA, and in mtRNA targeted

(g-h) HITC-CLIP read counts (y-axis) and its position on tRNA MT-TM, MT-TL1 and MT-TS2 (x-axis) for an unrelated control (ENSG00000182362) (g) and for NSUN3 (h)

(i) Structure and sequence of MT-TM with the HITS-CLIP targeted region in grey.

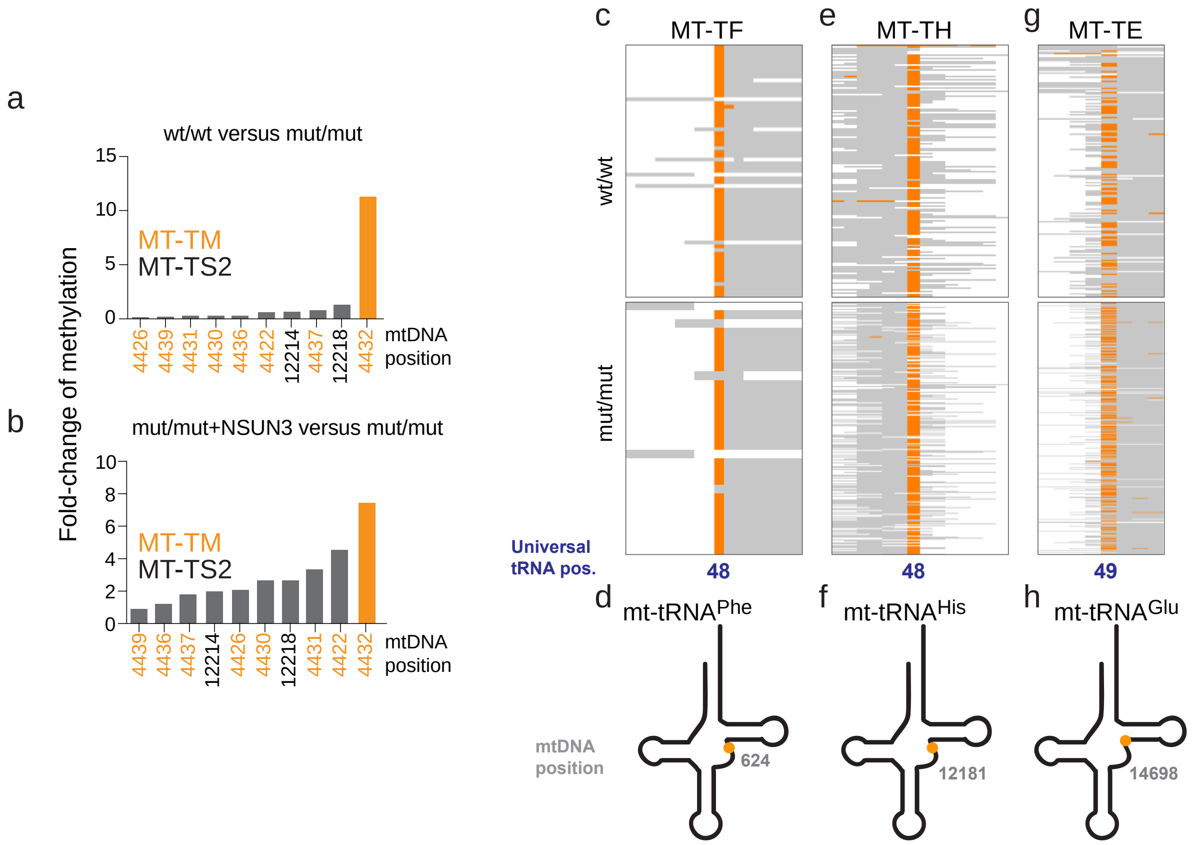

Supplementary Fig. 3:
(a-b) Fold-change of methylation at miCLIP sites (+/- 5 nucleotides) in mitochondrially encoded tRNAs in wild-type dermal fibroblast (wt/wt) (a) and rescued patient fibroblast (mut/mut+NSUN3) (b) versus fibroblasts lacking NSun3 (mut/mut) (see Tab. S2).
(c-e) Methylated (orange) and unmethylated (grey) cytosines (x-axis) and reads (y-axis) in wt/wt and mut/mut in MT-TF (c), MT-TH (d) and MT-TE (e).
(f-h) Structure and position of m^5C (orange circle) in MT-TF (f), MT-TH (g) and MT-TE (h)

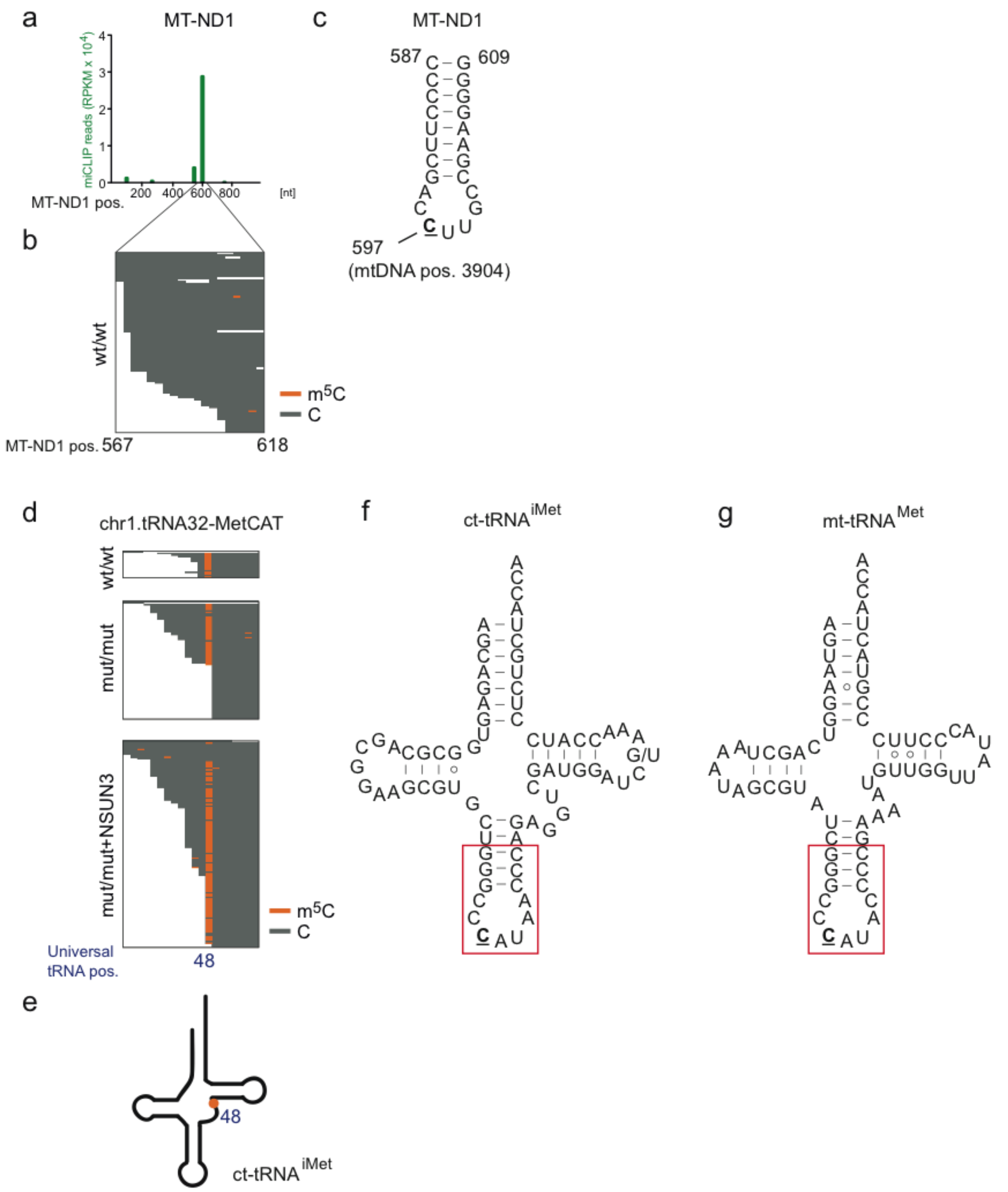

Supplementary Fig. 4:
(a) miCLIP reads (green) corresponding to the MT-ND1 gene.
(b) Methylated (orange) and unmethylated (grey) cytosines (x-axis) detected by BS RNA-Seq (individual reads on y-axis) for the region 567 - 618 of MT-ND1 for wt/wt.
(c) Potential stem-loop structure in the MT-ND1 RNA (region 587-609). The cytosine at position equivalent to the universal tRNA position 34 is underlined.
(d) Methylated (orange) and unmethylated (grey) cytosines (x-axis) detected by BS RNA-Seq (individual reads on y-axis) for the cytoplasmic (ct-) $tRNA^{iMet}$ gene for wt/wt, mut/mut and mut/mut+NSUN3 cells.
(e) Structure and position of m^5C (orange circle) in ct- $tRNA^{iMet}$.
(f-g) The clover leaf structure and sequence of human ct-$tRNA^{iMet}$ (f) and mt-$tRNA^{Met}$ (g). The red box indicates the similarity in the anticodon loop between the two species. The cytosine at the universal tRNA position 34 is underlined.

a

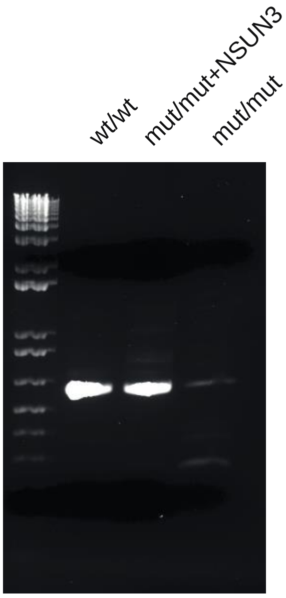

b

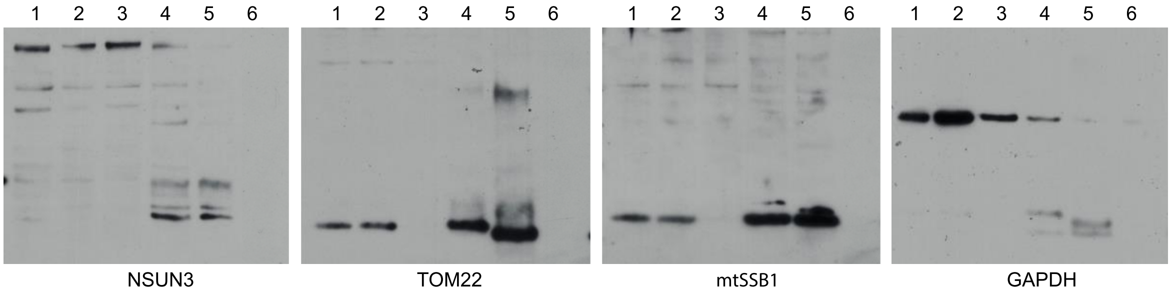

Supplementary Fig. 5:
(a) Source data - gel electrophoresis Fig. 1C. (b) Source data - western blots shown in Fig. 1g

a

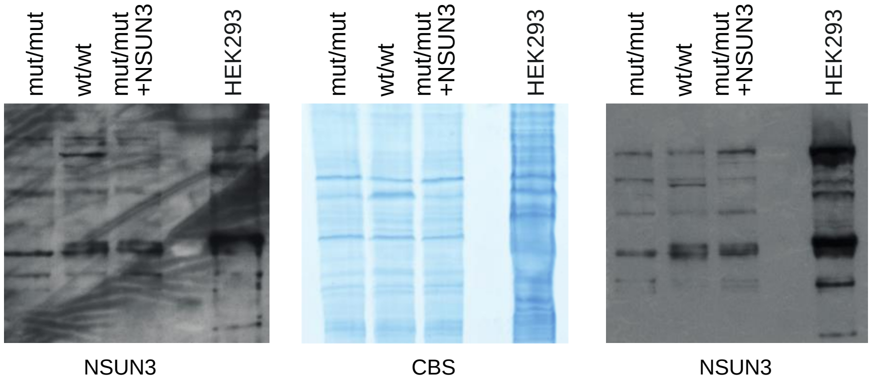

b

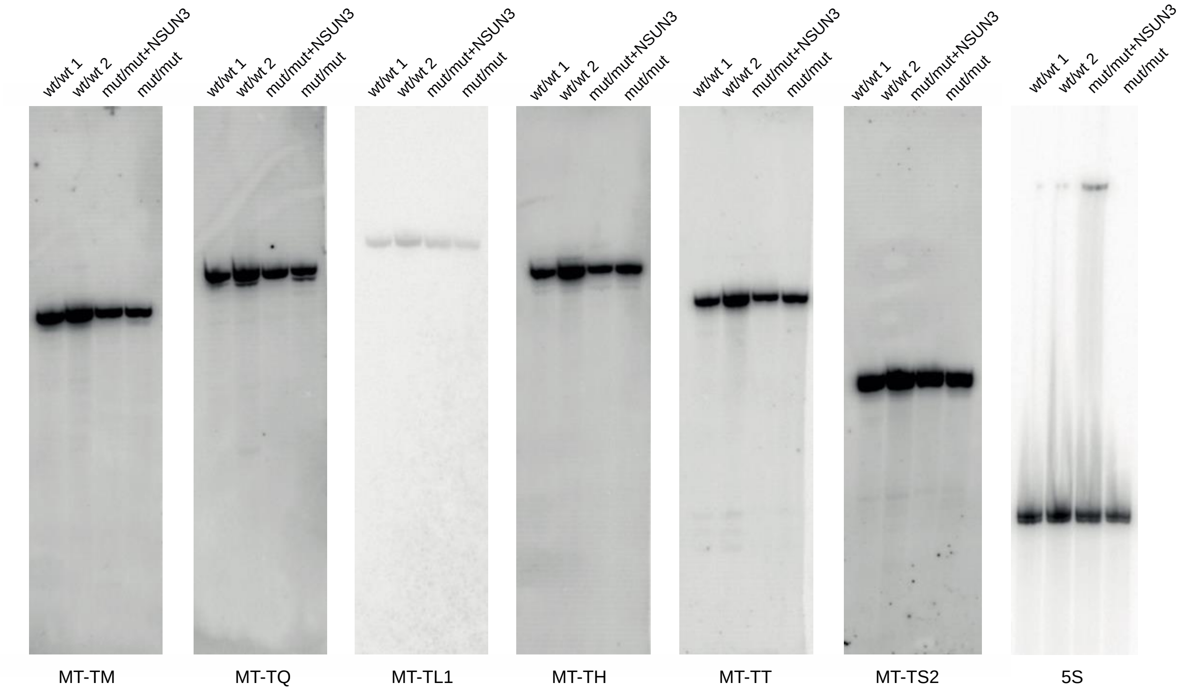

c

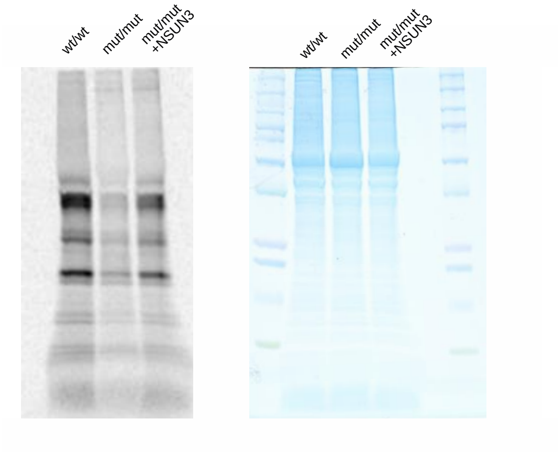

Supplementary Fig. 6:
(a) Source data - western blots shown in Fig. 2a. (b) Source data - northern blots shown in Fig. 2f. (c) Source data - 35S labelling shown in Fig. 2g

Gene	Human mtDNA position	Position (tRNAdb)	Location	Bovine	Human	Reference
MT-TF	624	48	Variable loop	not C	**m^5C**	
MT-TL1	3279	48	Variable loop	m^5C	m^5C	Suzuki 2014<br>Helm 1999
MT-TM	4432	34	Anticodon loop	f^5C	**m^5C**/f^5C	Suzuki 2014
MT-TW	5557	48	Variable loop	m^5C	not C	Suzuki 2014
MT-TN	5682	48	Variable loop	m^5C	not C	Suzuki 2014
MT-TH	12181	48	Variable loop	not C	**m^5C**	
MT-TS2	12239	48	Variable loop	n.d.	m^5C	Squires 2012
	12240	49	T-stem	m^5C	m^5C	Suzuki 2014<br>Squires 2012
	12241	50	T-stem	n.d.	m^5C	Squires 2012
MT-TE	14698	49	T-stem	m^5C	m^5C	Squires 2012
MT-TT	15952	72	Acc-stem	m^5C	n.d.	Suzuki 2014

n.d. – cysteine present, but methylation not detected.
Novel m^5C in human mt-tRNAs detected in this work are indicated in red.
Previously identified m^5C in human mt-tRNA and confirmed in this study are underlined

Supplementary Table 1: Cytosine-5 methylation in mammalian mitochondrial tRNA

NSun3 cDNA constructs		
miCLIP mutant		
forward		GTG TTA GTG GA TGC TCC GGC TTC AAA TGA TCG AAG CTG G
reverse		CCA GCT TCG ATC ATT TGA AGC CGG AGC ATC CAC TAA CAC
Overexpression in human mut/mut fibroblasts and cloning into pLenti6,3/V5-TOPO		
forward		CGC CAT GCT GAC CCA GCT GAA AG
reverse		TCA CCA TTT TCC TGT GCT CCA TG
Nsun3-V5 forward		CGC CAT GCT GAC CCA GCT GAA AG
NSun3-V5 reverse		CTA CGT AGA ATC GAG ACC GAG
Overexpression in HeLa cells: cloning into pcDNA5-FST2		
forward		CAG ACG GTA CCA TGC TGA CCC AGC TGA AAG C
reverse		ACG TCC TCG AGT TTT CCT GTG CTC CAT GAT T
mtDNA copy number determination		
mtDNA forward		CAC CCA AGA ACA GGG TTT GT
mtDNA reverse		TGG CCA TGG GTA TGT TGT TAA
mtDNA probe		6-Fam-TTA CCG GGC TCT GCC ATC T-Tamra
Genomic (B2M) forward		TGC TGT CTC CAT GTT TGA TGT ATC T
Genomic (B2M) reverse		TCT CTG CTC CCC ACC TCT AAG T
Genomic (B2M) probe		6-Fam-TTG CTC CAC AGG TAG CTC TAG GAG G-Tamra
Deletion analysis		
DNA		
PCR forward		TAG GCA CAT GAT GCA AGC CA
PCR reverse		GAG GGA CAT AGC CTT CAG CC
Sanger sequencing		GAT GTA CTG CAG GGA GGG ACT AA
RNA (PCR and Sanger sequencing)		
forward		CAG AGG GGA AGC TTG CAA AAC
reverse		ACA CGG AGC ATC CAC TAA CA
Mitochondrial RNA analysis		
GAPDH	forward	GAA GGT GAA GGT CGG AGT CAA C
	reverse	CAG AGT TAA AAG CAG CCC TGG T
	probe	6-Fam-GTT TGG TCC GTA TTG GGC GCC T-Tamra
CO1	forward	CGA TGC ATA CAC CAC ATG AA
	reverse	AGC GAA GGC TTC TCA AAT CA
	probe	6-Fam-GGC TCA TTC ATT TCT CTA ACA GC-Tamra
CO2	forward	CGT CTG AAC TAT CCT GCC CG
	reverse	TGG TAA GGG AGG GAT CGT TG
	probe	6-Fam-CGC CCT CCC ATC CCT ACG CAT C-Tamra
16S rRNA	forward	TTT GCA AGG AGA GCC AAA GC
	reverse	AGA CGG GTG TGC TCT TTT AGC T
	probe	6-Fam-AGA CCC CCG AAA CCA GAC GAG CTA CC-Tamra
12S rRNA	forward	CCC CAG GTT GGT CAA TTT C
	reverse	CGG CTT CTA TGA CTT GGG TTA A
	probe	6-Fam-TGC AGC CAC CGC GGT CA-Tamra

Supplementary Table 2: Sequences of primers and probes

Supplementary Note 1:

Clinical and Biochemical Characterisation of the Patient

Patient

Mitochondrial disorder, combined developmental disability, microcephaly, failure to thrive, recurrent incresed lactate levels in plasma, muscular weakness, proximal accentuated, external ophthalmoplegia, convergence nystagmus

Age of onset: 3. Month of life

Muscle histochemistry and respiratory chain analyses

The functional analysis of unfrozen muscle biopsy by radiochemical substrate oxidation (see Table 1) demonstrated normal protein activities. The ratio of the reactions forming considerable amounts of reduction equivalents (e.g. Pyruvate+Malate/Pyruvate+Carnitine) was slightly suspicious. The isolated measurement of the respiratory chain complexes (see Table 2) revealed a noticeable decrease in cytochrome c oxidase as well as a slight decrease in complex I and III. The reduction of complex IV was also seen in western blot analysis. The measurement of the relative mtDNA content using quantitative real-time PCR showed normal levels.

Substrate oxidation of the 600g fraction of unfrozen muscle homogenate										
**[nmol/h/mg protein]**			*Normal range*			**[nmol/h/mUnit CS]**		*Normal range*		
[1-14C]Pyruvate+Malate	**579**		*263*	-	*900*	P+M/CS	**1,96**	*1,54*	-	*3,55*
[1-14C]Pyruvate+Carnitine	**779**		*302*	-	*856*	P+C/CS	**2,64**	*1,65*	-	*3,66*
[1-14C]Pyruvate+Malate-ADP	**104**		*32*	-	*102*	P+M- S	**0,35**	*0,21*	-	*0,41*
[U-14C]Malate+Pyruvate+Malonate	**665**		*282*	-	*874*	M+P+Mal/C	**2,25**	*1,56*	-	*3,87*
[U-14C]Malate+Acetylcarn.+Malonate	**784**	↑	*273*	-	*678*	M+AC+Mal/	**2,66**	*1,16*	-	*2,82*
[U-14C]Malate+Acetylcarn.+Arsenite	**428**	↑	*156*	-	*378*	M+AC+As/C	**1,45**	*0,57*	-	*1,52*
[U-14C]Glutamate+Acetylcarnitine	**269**	↑	*86*	-	*209*	G+AC/CS	**0,91**	*0,35*	-	*1,06*

Enzyme measurements of the 600g fraction of unfrozen muscle homogenate											
**[nmol/h/mg protein]**			*Normal range*			**[nmol/h/mUnit CS]**			*Normal range*		
Citrate synthase (CS)	**295**		*150*	-	*338*						
Complex I (CI)	**26**		*28*	-	*76*	CI/CS	**0,09**	↓	*0,14*	-	*0,35*
Complex I+III (C13)	**114**		*49*	-	*218*	C13/CS	**0,38**		*0,24*	-	*0,81*
Complex II (CII)	**71**		*33*	-	*102*	CII/CS	**0,24**		*0,18*	-	*0,41*
Complex II+III (C23)	**153**		*65*	-	*180*	C23/CS	**0,52**		*0,30*	-	*0,67*
Complex III (CIII)	**220**	↓	*304*	-	*896*	CIII/CS	**0,74**	↓	*1,45*	-	*3,76*
Cytochrom c Oxidase (COX)	**118**	↓	*181*	-	*593*	COX/CS	**0,40**	↓	*0,91*	-	*2,24*
Complex V (CV)	**230**		*86*	-	*257*	CV/CS	**0,78**		*0,42*	-	*1,26*
Pyruvate dehydrogenase (PDHC)	**21,0**		*5,3*	-	*19,8*	PDHC/CS	**0,071**		*0,026*	-	*0,07*

Supplementary References:

Jühling F, Mörl M, Hartmann RK, Sprinzl M, Stadler PF, and Pütz J. (2009) tRNAdb 2009: compilation of tRNA sequences and tRNA genes. *Nucleic Acids Res.*, **37**: Database issue: D159-D162.

Helm M, Florentz C, Chomyn A, Attardi G. (1999) Search for differences in post-transcriptional modification patterns of mitochondrial DNA-encoded wild-type and mutant human tRNALys and tRNALeu(UUR). *Nucleic Acids Res*. **27**:756-63.

Squires JE, Patel HR, Nousch M, Sibbritt T, Humphreys DT, Parker BJ, Suter, CM and Preiss T. (2012) Widespread occurrence of 5-methylcytosine in human coding and non-coding RNA. *Nucleic Acids Res*. **40**: 5023–5033.

Suzuki T and Suzuki T. A complete landscape of post-transcriptional modifications in mammalian mitochondrial tRNAs. (2014) *Nucleic Acids Res*. **42**: 7346–7357.
